# Correction to: ‘Age determination in echinoderms: first evidence of annual growth rings in holothuroids’ 2022 by Sun *et al.*

**DOI:** 10.1098/rspb.2022.1872

**Published:** 2022-10-12

**Authors:** Jiamin Sun, Jean-François Hamel, Bruno L. Gianasi, Annie Mercier

*Proc. R. Soc. B* **286**, 20190858. (Published online 10 July 2019) (https://doi.org/10.1098/rspb.2019.0858)

The correction concerns figure 2, in which the sizing of panels (*b*) and (*d*) and the scale bar were not correct. The panels were resized in the revised figure, and the value of the scale bar adjusted to 1 mm instead of 200 µm. Changes to this figure and its caption do not impact any of the analyses, results, interpretations or conclusions.

**Figure 2.**
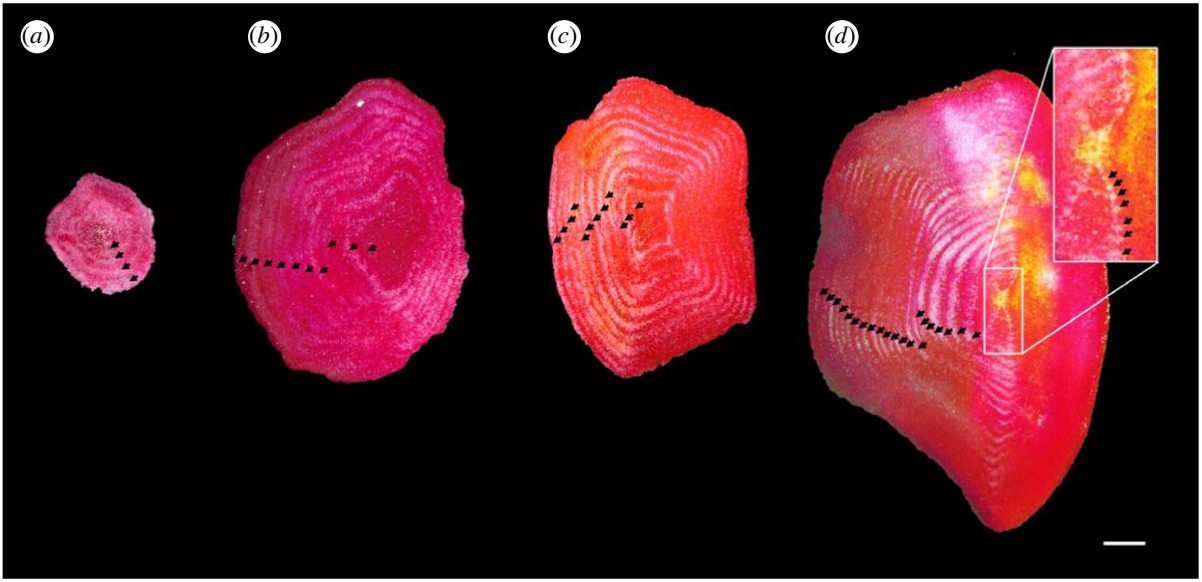
Plates from wild-caught individuals of *Psolus fabricii* of different sizes, with different numbers of growth rings: (*a*) 4 rings, (*b*) 10 rings, (*c*) 12 rings and (*d*) 28 rings. One pair, consisting of a dark and a light ring, was considered to represent 1 year of growth. Therefore, the innermost rings of individuals shown in (*a*), (*b*), (*c*) and (*d*) were, respectively, developed in 2013, 2007, 2005 and 1989 and all the outermost rings were developed in 2017. The scale bar at the bottom represents 1 mm. (Online version in colour.)

